# Household food insecurity and dietary patterns in rural and urban American Indian families with young children

**DOI:** 10.1186/s12889-017-4498-y

**Published:** 2017-06-30

**Authors:** Emily J. Tomayko, Kathryn L. Mosso, Kate A. Cronin, Lakeesha Carmichael, KyungMann Kim, Tassy Parker, Amy L. Yaroch, Alexandra K. Adams

**Affiliations:** 1 Nutrition, School of Biological and Population Health Sciences, College of Public Health and Human Sciences, Oregon State University, Corvallis, OR USA; 2 Department of Family Medicine and Community Health, School of Medicine and Public Health, University of Wisconsin, Madison, WI USA; 3 Department of Biostatistics and Medical Informatics, School of Medicine and Public Health, University of Wisconsin, Madison, WI USA; 4 Department of Family and Community Medicine, School of Medicine, University of New Mexico, Albuquerque, NM Mexico; 5 Gretchen Swanson Center for Nutrition, Omaha, NE USA; 6 Center for American Indian and Rural Health Equity, Montana State University, AJM Johnson Hall 103B, P.O. Box 173485, Bozeman, MT 59715 USA

**Keywords:** American Indian, Food security, Urban, Rural, Diet, Early childhood

## Abstract

**Background:**

High food insecurity has been demonstrated in rural American Indian households, but little is known about American Indian families in urban settings or the association of food insecurity with diet for these families. The purpose of this study was to examine the prevalence of food insecurity in American Indian households by urban-rural status, correlates of food insecurity in these households, and the relationship between food insecurity and diet in these households.

**Methods:**

Dyads consisting of an adult caregiver and a child (2–5 years old) from the same household in five urban and rural American Indian communities were included. Demographic information was collected, and food insecurity was assessed using two validated items from the USDA Household Food Security Survey. Factors associated with food insecurity were examined using logistic regression. Child and adult diets were assessed using food screeners. Coping strategies were assessed through focus group discussions. These cross-sectional baseline data were collected from 2/2013 through 4/2015 for the Healthy Children, Strong Families 2 randomized controlled trial of a healthy lifestyles intervention for American Indian families.

**Results:**

A high prevalence of food insecurity was determined (61%) and was associated with American Indian ethnicity, lower educational level, single adult households, WIC participation, and urban settings (*p* = 0.05). Food insecure adults had significantly lower intake of vegetables (*p* < 0.05) and higher intakes of fruit juice (<0.001), other sugar-sweetened beverages (*p* < 0.05), and fried potatoes (*p* < 0.001) than food secure adults. Food insecure children had significantly higher intakes of fried potatoes (*p* < 0.05), soda (*p* = 0.01), and sports drinks (*p* < 0.05). Focus group participants indicated different strategies were used by urban and rural households to address food insecurity.

**Conclusions:**

The prevalence of food insecurity in American Indian households in our sample is extremely high, and geographic designation may be an important contributing factor. Moreover, food insecurity had a significant negative influence on dietary intake for families. Understanding strategies employed by households may help inform future interventions to address food insecurity.

**Trial registration:**

(NCT01776255). Registered: January 16, 2013. Date of enrollment: February 6, 2013.

## Background

Food insecurity is a growing public health concern in the United States, particularly among young children. In October 2015, the American Academy of Pediatrics urged physicians to screen children for food insecurity in recognition of the adverse health outcomes associated with inadequate access to food [1]. American Indian families may be particularly vulnerable, as they experience higher rates of factors associated with food insecurity, including poverty [2], limited access to healthy foods [3–6], and decreased food sovereignty (i.e., “community-level food security”, which includes access to culturally appropriate food [7]). Data on food insecurity in American Indian families are lacking in national datasets, but food insecurity rates as high as 75% have been reported in smaller studies of tribal communities [8–10]. To date, most studies of food security among American Indian families have been tribe or reservation based studies [9–11] or studies of indigenous populations in other countries [12, 13]. However, rates of food insecurity are known to differ between urban and rural areas in the general US population [14], and ~78% of people who identify as American Indian report living outside of tribal-designated areas, which are predominately rural [15]. Therefore, it is imperative to consider differences that may exist in food insecurity among American Indians in urban and rural settings.

The presence of food insecurity may impact not only the quantity of available food but also diet intake patterns [16–18], with some evidence of this relationship in older American Indian children [10]. However, no studies to date have examined the relationship between household food insecurity and dietary intake in American Indian adults or in very young American Indian children, particularly within the same household. We sought to address these important gaps in the literature by evaluating the prevalence of food insecurity among American Indian households from both rural and urban communities and examining the association of food insecurity with diet patterns of both adults and young children (2–5 years) concurrently in these households. These data were collected as part of the Healthy Children Strong Families 2 (HCSF2) randomized controlled trial of a healthy lifestyle intervention for American Indian families nationwide. [19] We sought to address three main research questions:

i. What is the prevalence of food insecurity in American Indian households, and how did this differ by urban-rural status?
ii. What are the correlates of food insecurity in American Indian households?
iii. What is the relationship between food insecurity and diet in these households?

## Methods

### Participant recruitment and enrollment

Participants were recruited from four rural and one urban community in the United States as part of the Healthy Children, Strong Families 2 randomized controlled trial, a healthy lifestyle intervention for American Indian families with young children. The primary objective of HCSF2 is to determine the efficacy of a wellness toolkit in preventing and reducing obesity among American Indian primary caregivers and their children (ages 2–5 years). Senior members of the research team approached communities with whom they had previously worked or where they had close relationships with community members or wellness staff to participate as HCSF2 study sites. Participant inclusion criteria included enrolling a child between the ages of 2 and 5 years and a primary caregiver (e.g., mother, father, grandparent), the ability to travel to the local data collection site for study visits, and a valid mailing address. Exclusion criteria were minimal due to the communities’ value for inclusion in community programs and projects. Caregivers provided written informed consent for both themselves and the participating child, and human subjects approval was granted through the University of Wisconsin Institutional Review Board (protocol 2012–0578) and tribal institutional review boards, when requested by tribal administration. Data reported here were collected during the baseline visit for HCSF2, prior to starting the 2-year intervention.

### Measures

#### Medical history

A self-administered 40-item medical history survey was used to collect information on age, sex, ethnicity, education, household size, income, use of food assistance programs (e.g., WIC, school lunch program), and questions related to chronic disease risk. Adults also provided information on child age, sex, ethnicity, and other demographic factors (e.g., child birthweight). This survey was developed by the study team and has been used previously in this population [20].

#### Food insecurity

Food insecurity was assessed using two items from the United States Department of Agriculture (USDA) 18-item Household Food Security Survey: “*Within the past 12 months we were worried whether our food would run out before we got money to buy more*” and “*Within the past 12 months the food we bought just didn’t last and we didn’t have money to get more*”. An affirmative answer to either of these two questions indicates food insecurity with 97% sensitivity and 83% specificity, and the use of these two questions has been validated against the full survey for households with young children [21]. The two-question survey was chosen to minimize participant burden due to the number of measures and time to complete all the questionnaires included in the larger obesity prevention intervention trial. An additional question was added as a proxy measure to assess distance to stores (*How far do you go to obtain food?*) as an additional variable related to food access, which may impact the ability of a household to obtain adequate food.

#### Dietary intake

A validated diet screener based on the Dietary Screener Questionnaire [22] used in the National Health and Nutrition Examination Survey (2009–2010) was used for adults, and the validated child dietary screener based on questions contained in the 2010 National Youth Physical Activity and Nutrition Survey (documentation at http://www.cdc.gov/healthyyouth/yrbs/nypans.htm) was used for children. For both screeners, questions asked about intake over the previous 7 days in the following categories: fruit, vegetables, salad, potatoes, fried potatoes, pizza, 100% juice, soda, other sugar sweetened beverages (e.g., lemonade, sweetened tea, fruit punch, Kool-Aid), and milk.

### Anthropometrics

An electronic scale measured weight (Tanita Model BWB-800S, Tanita, Inc., Chicago, IL), which was assessed without shoes and in light clothing to the nearest 0.1 kg. Height was measured with a portable stadiometer to the nearest 0.1 cm (Seca Model 217, Seca, Inc., Hanover, MD). The average of two measurements was used for height and weight. Children’s heights and weights were converted to body mass index (BMI) percentiles [23], and adult BMI was calculated as kg/m^2^.

### Focus groups

Focus groups were conducted by trained staff at sites that had completed the intervention to expand our understanding of how geography and other social factors affect food insecurity for participating families and to discuss coping strategies employed by families. Focus group participants were recruited by mailed invitation letters from among participants who had completed the HCSF2 intervention, and 5–7 participants were recruited for each session. A topic guide was developed to ensure consistency among focus group facilitators and included questions (e.g., *Do people in the community share food when there are others in need?*) and follow-up probes (e.g., *How does that work?*). Topics included food access, food sharing practices, use of food assistance, and other strategies for obtaining adequate food. Data were audio recorded and transcribed by a transcription service and were supplemented by research staff notes taken during each session. Major themes were determined by inductive methods by three independent trained research staff.

### Statistical analysis

Descriptive statistics were used to estimate prevalence of food insecurity in our sample overall and by urban and rural status. Differences in household and adult and child characteristics between the urban and rural geographic regions were assessed using Fisher’s exact/chi-square tests and Wilcoxon rank-sum tests for dichotomous, ordinal and nominal categorical, and measurement data, respectively. Predictors of food insecurity were assessed using univariate and multivariate logistic regression models. Predictors included in the multivariate models were ethnicity, caregiver age, educational level, geographic designation (rural vs. urban), number of adults in household (single adult household vs. more than one adult), number of children in the household, work outside the home (yes vs. no), and WIC participation. Factors were included in the multivariate analysis if there was evidence of an association with food insecurity status in the univariate analyses (*p* ≤ 0.10) or the factors have been shown to be associated with food insecurity status in the literature (e.g. single adult household and number of children in the household). Weight status was not significantly associated with food insecurity status and therefore was not included in the model. Separate models then were run for urban and rural households that additionally included distance traveled to store. This variable was not included in the overall model as it was highly correlated with geographic designation. Daily intake frequencies of food items were calculated from adult and child diet screeners. Intake frequencies between food secure and insecure participants were compared using Wilcoxon rank-sum tests and are summarized using medians and interquartile ranges. For the logistic regression analysis results, odds ratios (OR) and 95% confidence intervals are presented. A significance level of 0.05 was used for all analyses without adjustment for multiplicity of testing. All analyses were conducted using STATA 14 (StataCorp, College Station, TX, USA) and validated using SAS 9.4 (SAS Inc., Cary, NC, USA).

## Results

### Prevalence of food insecurity

**Fig. 1:**
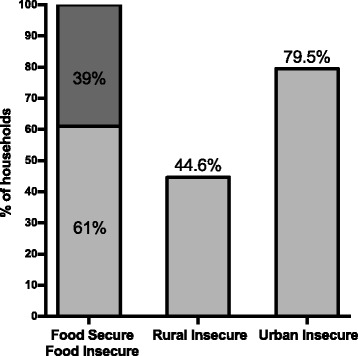
Prevalence of household food insecurity in the overall sample and by rural and urban status. Prevalence of household food insecurity was determined for the overall sample from Healthy Children, Strong Families 2 study (*n* = 450 households) and by rural (*n* = 240 households) and urban (*n* = 210 households) status using 2 validated questions from the USDA Household Food Security Survey

In total, 450 adult-child dyads from five participating communities (*n* = 240 rural households; *n* = 210 urban households) were enrolled. Participant demographics are summarized in Table 1. For adults, the average age was 31.5 ± 8.5 years, 95% were female, and 81.3% self-identified as American Indian; for children, average age was 45.0 ± 13.0 months, 50.0% were female, and 86.3% were identified by their caregiver as American Indian. The overall prevalence of food insecurity was 61% and was significantly higher in urban versus rural households at 80% versus 45%, respectively (*p* < 0.001, Fig. 1). Between food insecure and food secure households, significant differences were observed in education level (*p* < 0.01), income (*p* < 0.01), adult age (*p* < 0.05), and distance traveled to purchase food (*p* < 0.001).

**Table 1:** Household, adult, and child characteristics in overall sample and by urban and rural households for American Indian families with young children from five communities

	Overall (*n* = 450)	Geographic region		
		Urban (*n* = 210)	Rural (*n* = 240)	*p*-value
Household				
Food Insecure--*yes*, *n* (%)	267 (61.0)	163 (79.5)	104 (44.6)	<0.001
Education, *n* (%)				0.916
No College	169 (37.6)	81 (38.6)	88 (36.7)	
Some College/Associates Degree	235 (52.2)	108 (51.4)	127 (52.9)	
College degree or higher	46 (10.2)	21 (10.0)	25 (10.4)	
Income^a^, *n* (%)				<0.05
< $5000	132 (30.0)	72 (34.3)	60 (26.1)	
$5000–<$20,000	124 (28.2)	63 (30.0)	61 (26.5)	
$20,000–<$35,000	94 (21.4)	44 (21.0)	50 (21.7)	
≥ $35,000	90 (20.5)	31 (14.8)	59 (25.7)	
Number of children, median (IQR)	2 (2–3)	2 (1–3)	3 (2–3)	<0.01
Single adult household--yes, *n* (%)	106 (23.6)	50 (23.8)	56 (23.3)	0.906
Work outside the home--yes, *n* (%)	262 (58.2)	111 (52.9)	151 (62.9)	<0.05
WIC Participation^a^--yes, *n* (%)	357 (80.9)	177 (84.7)	180 (77.6)	0.058
Distance to store (miles), median (IQR)	5.0 (2.0–19.0)	2.2 (1.5–5.0)	15.0 (5.0–30.0)	<0.001
Adult				
Age (years), median (IQR)	30 (25–36)	29 (25–36)	30 (25–35)	0.933
Sex--female, *n* (%)	426 (94.7)	202 (96.2)	224 (93.3)	0.178
Ethnicity, *n* (%)				<0.001
American Indian	368 (81.8)	162 (77.1)	206 (85.8)	
White	42 (9.3)	11 (5.2)	31 (12.9)	
Hispanic	29 (6.4)	29 (13.8)	0 (0)	
Other	11 (2.4)	8 (3.8)	3 (1.3)	
Weight status, *n* (%)				<0.05
Normal	79 (18.7)	27 (13.5)	52 (23.3)	
Overweight	98 (23.2)	50 (25.0)	48 (21.5)	
Obese	246 (58.2)	123 (61.5)	123 (55.2)	
Child				
Age (months), median (IQR)	44.9 (34.1–55.4)	43.6 (31.5–54.4)	45.8 (36.4–57.5)	<0.05
Sex--female, *n* (%)	226 (50.2)	110 (52.4)	116 (48.3)	0.392
Ethnicity, *n* (%)				<0.001
American Indian	390 (86.7)	168 (80.0)	222 (92.5)	
White	24 (5.3)	7 (3.3)	17 (7.1)	
Hispanic	24 (5.3)	24 (11.4)	0 (0)	
Other	12 (2.7)	11 (5.2)	1 (0.4)	
Weight status, *n* (%)				<0.05
Normal	271 (60.4)	142 (67.6)	129 (54.0)	
Overweight	80 (17.8)	29 (13.8)	51 (21.3)	
Obese	98 (21.8)	39 (18.6)	59 (24.7)	

*IQR* interquartile range, *WIC* Special Supplemental Nutrition Program for Women, Infants, and Children

^a^Only 429 households had income and WIC participation data available

### Factors associated with food insecurity

Using logistic regression analysis, factors associated with food insecurity were assessed (Table 2). In the model that included all households, factors associated with significantly higher odds of food insecurity were adult ethnicity identified as American Indian (*p* < 0.05), WIC participation (*p* < 0.05), and urban households (*p* < 0.001), with a trend toward higher odds for single adult households (*p* = 0.054). Attainment of a college degree or higher was associated with significantly lower odds of food insecurity (*p* < 0.01). For rural households, single adult households were associated with significantly higher odds (*p* < 0.01), while attainment of a college degree or higher and working outside of the home were associated with lower odds of food insecurity (*p* < 0.05 for both). For urban families, the odds of food insecurity decreased with increasing distance traveled to purchase food (*p* < 0.05) and increased with an increasing number of children in the household (*p* < 0.05).

**Table 2:** Factors associated with food insecurity by logistic regression in American Indian families with young children for all households and by rural and urban households

	All (*n* = 450)		Rural (*n* = 240)		Urban (*n* = 210)	
	OR (95% CI)	*p*-value	OR (95% CI)	*p*-value	OR (95% CI)	*p*-value
Ethnicity (vs. *white*)						
American Indian	2.41 (1.12–5.19)	<0.05	2.14 (0.84–5.45)	0.11	2.63 (0.64–10.70)	0.18
Hispanic^a^	2.26 (0.66–7.71)	0.20			1.84 (0.35–9.61)	0.47
Other	2.16 (0.44–10.61)	0.34	2.98 (0.18–48.50)	0.44	1.92 (0.20–18.28)	0.57
Urban (yes)	4.78 (2.94–7.77)	<0.001				
Adult Age	0.98 (0.95–1.003)	0.09	0.98 (0.95–1.01)	0.22	0.97 (0.93–1.01)	0.20
Education (vs. No College)						
Some College	0.77 (0.47–1.25)	0.29	0.97 (0.51–1.84)	0.93	0.53 (0.23–1.25)	0.15
College degree +	0.31 (0.14–0.71)	<0.01	0.17 (0.03–0.85)	<0.05	0.35 (0.10–1.18)	0.09
Single adult household (yes)	1.68 (0.99–2.86)	0.05	2.72 (1.36–5.48)	<0.01	0.82 (0.35–1.88)	0.63
Number of children	1.03 (0.87–1.21)	0.76	0.92 (0.75–1.14)	0.46	1.43 (1.02–2.01)	<0.05
Work outside home (yes)	0.71 (0.45–1.13)	0.15	0.46 (0.25–0.86)	<0.05	1.33 (0.63–2.82)	0.46
WIC participation (yes)	1.89 (1.07–3.33)	<0.05	1.83 (0.83–4.03)	0.13	1.54 (0.62–3.84)	0.36
Distance to store			1.00 (0.99–1.01)	0.77	0.96 (0.92–0.99)	<0.05

*OR* odds ratio, *CI* confidence interval, *WIC* Special Supplemental Nutrition Program for Women, Infants, and Children. ^a^There were insufficient numbers of Hispanic participants from the rural households to include this category

### Dietary patterns among food insecure and food secure households

The frequency of daily intake of the following food groups was determined for both the adult and child: fruit, vegetables, salad, potatoes, fried potatoes, pizza, 100% juice, soda, other sugar sweetened beverages (SSBs, e.g., lemonade, sweetened tea, fruit punch, Kool-Aid), sports drinks, and milk (Table 3). Adults from food insecure households had significantly lower vegetable consumption (*p* < 0.05), and significantly higher intake of fried potatoes (*p* < 0.001), 100% fruit juice (*p* = 0.001), and other SSBs (*p* < 0.05). Children from food insecure households had significantly higher intake of salad (*p* < 0.01), fried potatoes (*p* < 0.05), soda (*p* = 0.01), and sports drinks (*p* < 0.05).

**Table 3:** Dietary intake for child and adult participants in food secure versus food insecure households in the overall sample and by urban/rural status

		Child [median (IQR)]			Adult [median (IQR)]		
		Food Secure (*n* = 171)	Food Insecure (*n* = 267)	*p*-value	Food Secure (*n* = 171)	Food Insecure (*n* = 267)	*p*-value
Fruit	Overall	1.00 (0.71–2.00)	1.00 (0.71–2.00)	NS	0.71 (0.29–1.00)	0.71 (0.29–1.00)	NS
	Rural	1.00 (0.71–2.00)	1.00 (0.71–2.00)	NS	0.71 (0.29–1.00)	0.71 (0.29–1.00)	NS
	Urban	0.86 (0.71–2.00)	1.00 (0.71–2.00)	NS	0.71 (0.29–2.00)	0.71 (0.29–1.00)	NS
Vegetables	Overall	0.71 (0.29–1.00)	0.71 (0.29–1.00)	NS	0.71 (0.29–1.00)	0.29 (0.29–0.71)	<0.05
	Rural	0.71 (0.29–1.00)	0.29 (0.29–0.86)	0.054	0.71 (0.29–1.00)	0.29 (0.29–0.71)	0.01
	Urban	0.71 (0.29–0.71)	0.71 (0.29–1.00)	NS	0.29 (0.29–1.00)	0.29 (0.29–1.00)	NS
Salad	Overall	0.29 (0.00–0.29)	0.29 (0.00–0.71)	<0.01	0.29 (0.29–0.71)	0.29 (0.29–0.71)	NS
	Rural	0.29 (0.00–0.29)	0.29 (0.00–0.29)	<0.05	0.29 (0.29–0.71)	0.29 (0.29–0.71)	NS
	Urban	0.29 (0.00–0.29)	0.29 (0.00–0.71)	NS	0.29 (0.29–0.71)	0.29 (0.29–0.71)	NS
Potatoes	Overall	0.29 (0.29–0.29)	0.29 (0.29–0.29)	NS	0.29 (0.29–0.29)	0.29 (0.29–0.29)	NS
	Rural	0.29 (0.29–0.71)	0.29 (0.29–0.71)	NS	0.29 (0.29–0.29)	0.29 (0.29–0.29)	NS
	Urban	0.29 (0.00–0.29)	0.29 (0.29–0.29)	<0.05	0.29 (0.29–0.29)	0.29 (0.29–0.29)	NS
Fried potatoes	Overall	0.29 (0.29–0.29)	0.29 (0.29–0.29)	<0.05	0.29 (0.29–0.29)	0.29 (0.29–0.71)	<0.001
	Rural	0.29 (0.29–0.29)	0.29 (0.29–0.29)	<0.05	0.29 (0.29–0.29)	0.29 (0.29–0.29)	<0.05
	Urban	0.29 (0.29–0.29)	0.29 (0.29–0.29)	NS	0.29 (0.29–0.29)	0.29 (0.29–0.71)	<0.01
Pizza	Overall	0.29 (0.29–0.29)	0.29 (0.29–0.29)	NS	0.29 (0.29–0.29)	0.29 (0.29–0.29)	NS
	Rural	0.29 (0.29–0.29)	0.29 (0.29–0.29)	NS	0.29 (0.29–0.29)	0.29 (0.29–0.29)	NS
	Urban	0.29 (0.29–0.29)	0.29 (0.29–0.29)	NS	0.29 (0.29–0.29)	0.29 (0.29–0.29)	NS
100% Juice	Overall	0.71 (0.29–1.00)	0.71 (0.29–2.00)	NS	0.29 (0.00–0.71)	0.29 (0.29–0.71)	0.001
	Rural	0.71 (0.29–1.00)	0.71 (0.29–1.00)	NS	0.29 (0.00–0.71)	0.29 (0.29–0.71)	<0.05
	Urban	0.71 (0.29–1.00)	0.71 (0.29–2.00)	NS	0.29 (0.00–0.29)	0.29 (0.29–0.71)	<0.05
Soda	Overall	0.00 (0.00–0.29)	0.29 (0.00–0.29)	0.01	0.29 (0.00–1.00)	0.29 (0.29–0.71)	NS
	Rural	0.00 (0.00–0.29)	0.29 (0.00–0.29)	NS	0.29 (0.00–1.00)	0.29 (0.29–1.00)	NS
	Urban	0.14 (0.00–0.29)	0.29 (0.00–0.29)	NS	0.29 (0.00–0.71)	0.29 (0.29–0.71)	NS
Other SSB	Overall	0.29 (0.00–0.29)	0.29 (0.00–0.71)	NS	0.29 (0.00–1.00)	0.71 (0.29–1.00)	<0.05
	Rural	0.29 (0.00–0.29)	0.29 (0.29–0.71)	NS	0.29 (0.00–1.00)	0.71 (0.29–1.50)	<0.05
	Urban	0.29 (0.00–0.29)	0.29 (0.00–0.71)	NS	0.29 (0.29–0.71)	0.29 (0.29–1.00)	NS

*IQR* interquartile range

Dietary patterns in food insecure and secure households were further analyzed by geographic status (urban and rural). Adults in rural food insecure households had lower intake of vegetables and higher intake of 100% fruit juice and SSBs compared to rural food secure households. For children from rural food insecure households, salad was significantly higher than their food secure counterparts. For adults in urban food insecure households, fried potatoes and 100% fruit juice were significantly higher compared to urban food secure households. Fried potatoes were also significantly higher for urban food insecure children compared to urban food secure households. For all food variables, adult and child mean daily intake was significantly correlated (*p* < 0.05), with two exceptions: adult and child intake of soda and milk was not significantly associated in rural food insecure households (data not shown).

### Focus groups

Six focus groups were held (two rural and one urban site) with a total of 31 adults between August 2015 and April 2016. Participants reported coping strategies employed during times of food insecurity, such as use of food assistance programs and relying on family members to supplement meals. Participants reported some intergenerational living or child care arrangements, which blunted some food insecurity through pooled resources but introduced a loss of parental control over some feeding choices. Some geographic differences in coping strategies were noted. For example, urban families with greater access to food outlets reported shopping frequently (every day or every other day), which resulted in spending more on food than planned. Rural families reported infrequent food purchasing trips, which often resulted in the purchase of fewer fresh fruits and vegetables. Rural families also reported using hunting, gathering, and sharing practices (e.g., hunting deer, harvesting wild rice) and individual/community gardens to supplement their diet. Table 4 includes sample comments from urban and rural participants.

**Table 4:** Sample comments from urban and rural focus group participants regarding food insecurity and coping strategies

Theme	URBAN (*n* = 16 participants among 3 sessions)	RURAL (*n* = 15 participants among 3 sessions)
Factors associated with shopping	On *more frequent* shopping: • “The majority of the time, I’m buying food for two days. I don’t go in and buy for a month. It’s just like, ‘this is what we need for tonight and tomorrow, and then we’ll figure it out from there’. But I feel like when I shop every two days I spend more money than if I knew what I was going to have for the week.” On shopping options: • “So you shop at different places, so you can get the best deals.” • “Lots of times, we’ll drive all over town looking for certain things at the best prices.”	On *less frequent* shopping: • “I guess toward the end of the month it’s a little hard, because fruit don’t last about two, three days in the house. But then the only times I get out is about once every two weeks.” • “And then your fruits and vegetables go bad really quick. Like if you buy lettuce you’re going to have to eat that right away---you won’t have that later on in the month.” • “I have to buy for two to three weeks at a time.”
Family sharing practices	• “We go to grandma’s house.” On loss of control: • “My mom and dad tend to give them what they want: soda, ice cream, doughnuts, candy, whatever it is.” • “Whatever, I don’t want my grandma feeding them Oreos for dessert, or dinner!”	• “I just go to my parents so I can go through their cupboards.” • “I go to my mom and dad’s freezer and go home with shopping bags.” On loss of control: • “When we eat at my mom’s, she’ll make comfort foods which aren’t always the healthiest, but at least I know my kids will eat.”
Use of food assistance programs	• “We go to the food pantry, and we get a food box from the school. There’s also a church that gives out food boxes.”	• “My kids get a lot of it [produce] from that gardening program at the Boys and Girls Club, so they help with the gardens around the community and they get sent home with whatever is ripe.” • “Our school district has snack packs that they send home and I think that helps a lot of parents too.”
Other coping strategies	• We will get a big, maybe, chicken and then when we get home we just immediately repackage so we get the whole thing out.” • “We buy stuff we know will keep, like boxes of cereal and pasta, like when you can get ten boxes of noodles for $10.”	“The times when we have money, I do big meals and then I freeze them.”
Reliance on local produce or bartering (rural only)		• “Summertime we have gardens, and we have friends who have gardens. So it’s kind of nice, we kind of do our exchange. Like my husband will fix their computer in exchange for something and then the ranching wife always has her garden and we do a lot of exchanges.” • “Where we live, we’re around farmers and stuff so there’s always chickens for eggs. And my mother in law, she’s into the whole canning and big gardens and stuff like that. • “People donate a lot of those zucchini, and there’s so many cucumbers”
Cost, Perceived Value, and Time (related to food choice)	• “You can eat healthier, but it’s so expensive, just for salad alone. You can have a budget, but for salad, it’s so expensive.” • “When you buy healthy foods it doesn’t seem to fill them up. You’ll feed them salad but you have to buy a lot of it in order for your kids to feel nourished.” • “Whatever is fast and quick, like frozen pizza.” • “I look in the papers for sales all the time.”	• “My biggest factor is the financials, the eating out thing. I don’t want to do it but it’s so much cheaper to just go out to eat then it is to buy things like fruit.” • “Fresh is more expensive, and doesn’t last.” • “When I’m tired and stressed, I just want whatever is going to get food on the table the fastest, and that’s usually a frozen pizza or something easy like that.”

## Discussion

An extremely high prevalence of food insecurity was identified among the American Indian households included in this study, and the proportion of households self-reporting food insecurity was significantly different between rural and urban households. These findings are significant as this is the first study of food insecurity to include both urban and rural American Indian families and to examine adults and children concurrently. Our analysis suggested identification as American Indian, urban households, lower educational levels, single adult households, and participation in WIC as factors that are associated with the increased odds for food insecurity. Moreover, differences in dietary intake patterns of both adults and children were identified between food insecure and secure households, suggesting the food insecurity negatively impacts dietary quality for these families. Different coping strategies were reported by rural and urban families that provide context to the quantitative findings.

The prevalence of food insecurity was significantly higher among urban households compared to their rural counterparts, which is of particular importance, as the majority of people who identify as American Indian report living outside of tribal-designated areas [15]. Two previous studies included urban American Indians in their sample, and neither found differences in food security between urban and rural participants. Gundersen examined a national sample of American Indian households (*n* = 1143) included in the 2001–2004 Core Food Security Module of the Current Population Survey and reported no interaction between food insecurity and geographic designation (i.e., rural or urban) for households with children [3]. The author did suggest these results were counterintuitive, as protective factors are known to exist within rural or reservation-based communities, including increased social capital [24] and food sharing practices [10, 25]. However, the extent to which these conditions may have contributed to the lower food insecurity in the rural communities in our study is unclear. Jernigan et al. examined food insecurity in a sample of low-income American Indians in California [26]. Of note, their study population was approximately half male and half Hispanic. In comparison, our sample included a full range of incomes, almost all females (~95%), and less than 10% Hispanic participants, and these demographic differences may have contributed to the differences in our findings.

Our analyses did suggest differences between rural and urban households with regard to factors associated with a higher risk of food insecurity. Namely, single adult households, lower educational attainment, and working outside of the home were associated with a high prevalence of food insecurity in rural households, while only the distance traveled to purchase food and the number of children in the household were associated factors in urban households. Our findings align with previous reports suggesting that identification as American Indian [3], not being employed outside of the home [8, 10], low education [8] and number of children in the household [9] are associated with a high prevalence of food insecurity in American Indian communities; findings related to the relationship between food security and participation in food assistance programs for American Indian have been conflicting [8]. As for distance traveled to purchase food, Mullany et al. demonstrated that households with transportation barriers were more likely to be food insecure [9], which may partially explain why increasing distance traveled to purchase food was associated with lower odds of food insecurity among urban households. In other words, these households may have more resources to travel greater distances to access food at lower cost, such as at Wal-Mart®, rather than relying on convenience stores and small markets where prices are typically much greater and the availability of fresh foods is limited.

Our findings demonstrated significant differences in dietary intake between food secure and insecure households for both American Indian adults and children. Bauer et al. found American Indian children (ages 5–6 years) from a rural reservation who were food insecure consumed more hot or ready-made food from convenience stores, including higher intake of pizza and fried chicken [10]. Adult diet was not considered, and no other studies have examined the relationship between food security status and dietary intake patterns in American Indian families. Moreover, we demonstrated differences among dietary intake for food insecure children compared to food secure children in very young children (2–5 years), which have not been previously demonstrated. These findings may be of clinical significance, as the food groups in which we identified differences in dietary intake patterns are known to contribute to obesity, such as high intake of fruit juice, soda, other SSBs, fried potatoes, and lower intake of vegetables. The finding of significantly increased salad intake among insecure children was unexpected, and we are seeking to better understand how adults are defining salad intake for their children.

### Strengths and limitations

This study was strengthened by the inclusion of both urban and rural American Indian households, as the majority of food security studies in American Indian populations to date have included only rural communities or single reservations [8–10, 27]. This factor also represents a potential limitation as our data were pooled from multiple, diverse communities. As another strength, our data are contextualized by findings from focus group sessions. For this study, we used only two items of the USDA 18-item Household Food Security Survey, which may have prevented us from capturing more nuanced dimensions of food security and may contribute to differences between our study and existing reports that used different measures [3]. However, these two particular survey items have been validated specifically in households with young children. The time of year when the food insecurity screener is administered may impact the responses, but this potential limitation likely was mitigated in our study as responses were collected over a 2-year period due to staggered enrollment at study sites. All of the survey measures used in the study were self-report, which may be associated with under- or over-reporting.

### Community responses

Many of the communities who participated in this study are currently drawing on traditional culture, strengths, and community resiliency to overcome existing barriers to food security, and we feel it is in alignment with community-based participatory research approaches to address their efforts here. For example, one participating community recently initiated a survey where 73% of respondents viewed hunger as an issue on the reservation [28]. Respondents suggested approaches to address food insecurity, namely, better coordination among programs, greater outreach to children and Elders, more jobs, adoption of a food sovereignty policy by the tribal legislature, and provision of classes (e.g., gardening, harvesting, wild game preparation, canning) and identified existing resources, including community-supported orchards/gardens, food distribution programs, and school-based feeding programs. Another participating rural community supports a strong land reclamation program emphasizing production of traditional foods (e.g., wild rice) in addition to food distribution programs and efforts focused on child and Elder nutrition. For families in the urban community in our study, several resources are provided through the health clinic, such as access to a community garden and food pantry, transportation passes to increase mobility to supermarkets with a broader range of options and prices, and a comprehensive community resource guide (updated monthly). The clinic also provides patient navigation services, including assistance meeting eligibility requirements for food assistance and services specific to American Indian families, such as obtaining a Certificate of Indian Blood and other tribal identification. This identification is needed for access to services like emergency funds provided by some tribes for their citizens living in urban settings, discounts at various Indian-owned businesses, and school-based American Indian-specific programs.

## Conclusions

Our data suggest food insecurity among American Indian households was extremely high and was significantly higher in urban households. Moreover, less optimal dietary patterns were identified for food insecure families in both urban and rural settings. Interventions to address food insecurity are urgently needed and must consider multiple factors related to food security, such as barriers that may be unique to families in urban settings (e.g., absence of close-knit community) and access to healthy food (e.g., reliance on small markets where prices are higher).

In addition, development of tribal programs and policies to address food insecurity, food sovereignty, and food access may supplement federal-level food assistance strategies; cultural factors unique to American Indian communities should be considered.

## Abbreviations

BMI: Body mass index
HCSF2: Healthy Children, Strong Families 2
IQR: Interquartile range
OR: Odds ratio
SSB: Sugar sweetened beverage
USDA: United States Department of Agriculture
WIC: Special Supplemental Nutrition Program for Women, Infants, and Children
